# Management of neovascular glaucoma with intravitreal ranibizumab, panretinal photocoagulation, and subsequent 5-fluorouracil augmented trabeculectomy: A case report: Erratum

**DOI:** 10.1097/MD.0000000000008130

**Published:** 2017-09-15

In the article, “Management of neovascular glaucoma with intravitreal ranibizumab, panretinal photocoagulation, and subsequent 5-fluorouracil augmented trabeculectomy: A case report”,^[1]^ which appeared in Volume 96, Issue 25 of *Medicine*, the authors’ affiliation should have appeared as “"Department of Ophthalmology, Union Hospital, Tongji Medical College, Huazhong University of Science and Technology, Wuhan 430022, China.”
